# Snoring and obstructive sleep apnoea as risk factors in SARS-Cov-2: can nasal CPAP during sleep reduce pneumonia risk?

**DOI:** 10.1007/s41105-020-00295-5

**Published:** 2020-11-07

**Authors:** Colin E. Sullivan

**Affiliations:** Professor Emeritus, University of Sydney, Sydney, Australia

Old age, hypertension, obesity and diabetes are the major risk factors for severe disease with COVID 19. However, snoring and obstructive sleep apnoea (OSA), unrecognised, or under reported [1], occurs in over 50% of these comorbidities [2], and is a known risk factor for pneumonia. Preliminary reports confirm an association between OSA and severe COVID 19 disease [3].

Aspiration during sleep is among several pathways by which snoring, and sleep apnoea increases vulnerability to pneumonia [4]. The propensity for aspiration increases with age related deterioration in pharyngeal competence. Apnoeic events, and the vibrating pharyngeal airway of snoring, coupled with strong inspiratory efforts, are powerful mechanisms capable of sucking large amount of viral loaded saliva and mucus from the nasopharyngeal airway into the lower airways and lungs. The early reports of patchy non-uniform pneumonic changes in COVID 19 is highly suggestive of aspiration. In an elegant study, Hou et al. [5] provide evidence for aspiration as a major mechanism leading to viral pneumonia.

While some have recommended suspending the use of nasal positive pressure therapy (CPAP) and non-invasive positive pressure ventilation (NIV) because of the risk of viral spread [6], CPAP with oxygen via a face mask or helmet has become a major part of the management of the COVID respiratory failure, with reports suggesting that many pneumonia patients avoid full intubation [7, 8]. In those who already have developed pneumonia, the benefit of the positive pressure is in its role in maintaining alveolar air spaces for gas exchange, helping to prevent airway closure and alveolar loss.

However, another, perhaps more important benefit of CPAP use in sleep, is preventing aspiration, by stopping snoring and apnoea. In the time window between the initial viral infection in the nasopharynx and the onset of the lung disease, unrecognised snoring and sleep apnoea may be significant mechanisms increasing aspiration during sleep and exposing the lungs to high levels of viral inoculates. Rather than waiting for the patient to develop lung changes, early use of nasal CPAP in sleep might offer a safe method of helping to reduce the progression to potentially fatal COVID 19 pneumonia. The report by Oranger et al. [8] supports this idea, as CPAP was used continuously only during the night, and thus during presumed sleep. The problem of viral spread can be minimized with the use of appropriate circuit filters [8, 9, 10], good mask fit [11, 2] including the use of adapted surgical masks over a nasal CPAP interface [13, 14], and usual barrier nursing. Given that mask CPAP is being used to help slow the progress of the worsening pneumonia once respiratory failure has developed, introducing it earlier to the sleeping patient with COVID 19 does not require a major change in clinical protocols.
